# Complete chloroplast genome of the oil-bearing shrub *Staphylea bumalda* DC (Staphyleaceae)

**DOI:** 10.1080/23802359.2022.2164701

**Published:** 2023-01-13

**Authors:** Han Zhao, Yue Qin, Gaopu Zhu, Tao Han, Panfeng Liu

**Affiliations:** a Research Institute of Non-Timber Forestry, Chinese Academy of Forestry, Zhengzhou, PR China; b State-Owned Tongbai Maoji Forest Farm Tongbai, PR China

**Keywords:** *Staphylea bumalda*, chloroplast genome

## Abstract

*Staphylea bumalda* DC, belonging to family Staphyleaceae, is a woody understory tree that is both edible and medicinal and produces oil with high economic value. This study reports the first complete chloroplast genome sequence of *S. bumalda*. The complete chloroplast genome sequence of *S. bumalda* is 160,319 bp in length with an overall GC content of 32.79%, which is composed of a large single-copy region (LSC: 89,401 bp), a small single-copy region (SSC: 18,834 bp), and two inverted repeat regions (IR: 26,042 bp). A total of 130 genes were predicted in this genome, including 85 protein-coding genes, 37 tRNA genes, and eight rRNA genes. The phylogenetic analysis based on 14 complete chloroplast sequences from related species revealed that *S. bumalda* is a sister to *S. holocarpa*.

*Staphylea bumalda* (Candolle 1825) is a shrub species in family Staphyleaceae mainly distributed throughout China, Japan, and Korea. Also called pearl flower, its floral buds contain a high content of crude protein, crude fat, and essential amino acids and are consumed as a vegetable in the mountainous regions of China for their unique flavor (Yang et al. 2013).The root of *S*. bumalda is used in Traditional Chinese Medicine for clearing heat and detoxification as well as reducing swelling, dispelling masses, moisturizing the lungs and cough suppression (Song 1999). Moreover, the seed oil yield is 32%, which is comparable to that of woody oil tree species such as hazelnut and walnut (Liu et al. 2008), which can be used to develop functional vegetable oils (Li et al. 2007). However, there are still few studies on the phylogenetic relationships among *S. blumalda* and related taxa. Here, we characterized the complete chloroplast genome sequence of *S*. bumalda using high throughput sequencing technology to provide a resource for exploring evolutionary relationships in Staphylea.

The fresh leaves of *S. bumalda* were collected from the State-owned Tongbai Maoji Forest Farm (32°23′45″N, 113°41′32″E) of Henan Province, China. The voucher specimen is deposited in the National Center for Forestry and Grassland Genetic Resources (contact person: Han Zhao, Hanzhao@caf.ac.cn; specimen code 1111C0003307005096). Total genomic DNA was extracted according to a modified CTAB protocol (Doyle and Doyle 1987). The total DNA quantity and purity were analyzed using Bioanalyzer 2100 and RNA 6000 Nano LabChip Kit (Agilent, San Diego, CA, USA).The library was constructed with an insert length of 300 bp (±50 bp) and the genome sequencing was performed by llumina Hiseq 2500 Platform (San Diego, CA, USA). After removing low-quality reads and trimming adapter sequences, approximately 1.2 GB of high-quality clean reads remained. The filtered reads were assembled by SOAPdenovo (version: 2.04, http://soap.genomics.org.cn/soapdenovo.html) short sequence assembly software (Luo et al. 2015). The inner hole of the assembly result were repaired by GapCloser (version: 1.12, http://soap.genomics.org.cn/soapdenovo.html) software. The genome was annotated using the CpGAVAS2 pipeline (Shi et al. 2019), and adjusted using Geneious 20.2.2 (http://www.geneious.com). The new annotated chloroplast genome sequence was deposited in GenBank (Accession No. CRA005805).

The chloroplast genome sequence of *S. bumalda* was 160,319 bp in length, consisting of a small single-copy region (SSC: 18,834 bp), a large single-copy region (LSC: 89,401bp), and two inverted repeat regions (IR: 26,042 bp), with the overall GC content of 32.79%. The chloroplast genome encoded a total of 130 genes, including 85 protein-coding genes, 37 tRNA genes, and eight rRNA genes. Twenty-seven introns were distributed across twenty-one genes contained. The gene content of the *S. bumalda* cp genome was nearly identical to those of related taxa. However, two genes, *LhbA* and *InfA* were lost in *S. bumalda* compared with other species.

To explore the phylogenetic relationships within tree species of related families and genera, a maximum likelihood phylogeny analysis was performed using IQ-TREE 2.1.3 with 1000 bootstrap replicates (Nguyen et al. 2015; Minh et al. 2020). A total of 14 species were used, including seven Staphyleaceae species, five *Sambucus* species, one *Bischofia* species, and one *Acer lucidum* species as outgroup. Genome sequences were downloaded from the GenBank database and were aligned using MAFFT v7.0 (Katoh and Standley 2013). As shown in the phylogenetic tree (Figure 1), the fourteen species were organized into five clusters. The species of Staphyleaceae and other families each formed separate clades, including those historically considered related to Staphylea. Staphyleaceae species have a relatively high degree of genetic similarity, and *S. bumalda* was sister to *S*. holocarpa. The topology shows *Tapiscia sinesis* is distantly related from *S. blualda* and other Staphylea. This supports previous research describing the unique characters of Tapiscia that warrant its treatment as a separate family (Ding and Yu 1992; Shida and Tokuoka 2017).

**Figure 1.**
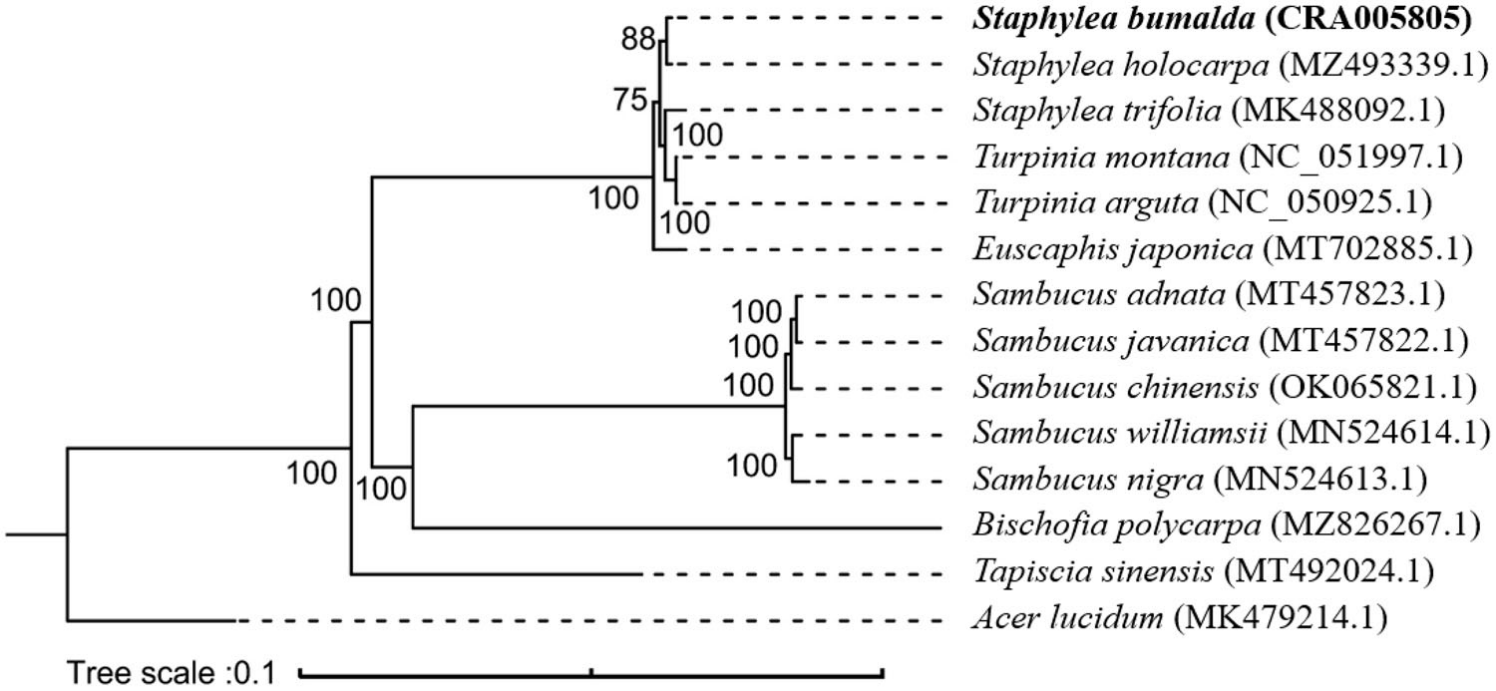
The maximum likelihood reconstruction of *Staphylea bumalda* and related species showing a monophyletic Staphyleaceae separate from taxa once considered related to *Staphylea*. Bootstrap values (*N* = 1000) are indicated at each node.

## Data Availability

The genome sequence data that support the findings of this study are openly available in GenBank of CNCB at [https://ngdc.cncb.ac.cn] (https://ngdc.cncb.ac.cn) under the accession no. PRJCA007887. The associated BioProject, SRA, and Bio-Sample numbers are PRJCA007887, CRA005804, and SAMC545073 respectively.
